# Caloric Restriction in Group-Housed Mice: Littermate and Sex Influence on Behavioral and Hormonal Data

**DOI:** 10.3389/fvets.2021.639187

**Published:** 2021-04-15

**Authors:** Cristina Perea, Ana Vázquez-Ágredos, Leandro Ruiz-Leyva, Ignacio Morón, Jesús Martín Zúñiga, Cruz Miguel Cendán

**Affiliations:** ^1^Center of Scientific Instrumentation, University of Granada, Granada, Spain; ^2^Department of Psychobiology, Institute of Neurosciences, Center for Biomedical Research (CIBM), University of Granada, Granada, Spain; ^3^Department of Pharmacology, Faculty of Medicine, Biomedical Research Center, Institute of Neuroscience, University of Granada, Parque Tecnológico de Ciencias de la Salud, Granada, Spain; ^4^Biosanitary Research Institute ibs.GRANADA, Granada, Spain; ^5^Department of Psychobiology, Faculty of Psychology, Center of Investigation of Mind, Brain, and Behavior, University of Granada, Granada, Spain

**Keywords:** caloric restriction, grouped mice, littermate mice, adrenocorticotropic, eating behavior, social behavior

## Abstract

Much of the research done on aging, oxidative stress, anxiety, and cognitive and social behavior in rodents has focused on caloric restriction (CR). This often involves several days of single housing, which can cause numerous logistical problems, as well as cognitive and social dysfunctions. Previous results in our laboratory showed the viability of long-term CR in grouped rats. Our research has studied the possibility of CR in grouped female and male littermates and unrelated CB6F1/J (C57BL/6J × BALBc/J hybrid strain) mice, measuring: (i) possible differences in body mass proportions between mice in *ad libitum* and CR conditions (at 70% of *ad libitum*), (ii) aggressive behavior, using the number of *pushes* and *chasing behavior time* as an indicator and social behavior using the *time under the feeder* as indicator, and (iii) difference in serum adrenocorticotropic hormone (ACTH) concentrations (stress biomarker), under *ad libitum* and CR conditions. Results showed the impossibility of implementing CR in unrelated male mice. In all other groups, CR was possible, with a less aggressive behavior (measured only with the number of *pushes*) observed in the unrelated female mice under CR conditions. In that sense, the ACTH levels measured on the last day of CR showed no difference in stress levels. These results indicate that implementantion of long-term CR in mice can be optimized technically and also related to their well-being by grouping animals, in particular, related mice.

## Introduction

Caloric restriction (CR) has been widely used in experimental research (1, 2). CR has been used in different modalities (moderate, 70–80% and intense restriction, 50–60%), with respect to the maximum *ad libitum* (AL) intake. It is proposed as a maintenance method between 6 and 24 months or more (3). Overfeeding is considered one of the most uncontrolled variables in bioassays in general (4). The most commonly used in chronic (24 months) and/or subchronic (12 months) evaluation studies is the moderate (70–80%) CR procedure (4–7). Traditionally, CR research has focused on how it influences increased longevity (2, 8, 9). CR has also been studied in relation to oxidative stress, where it was found to have an antioxidant effect (10), or in relation to the reduction of inflammatory processes induced by aging and measured in microglia levels (11). Another context in which CR has been studied is anxiety. Thus, CR has proven to have an anxiolytic effect, tested in the open field and in elevated plus maze (12, 13) and it enhances fear extinction learning (14), but has no effect on post-traumatic stress disorder (15). CR research also studies cognitive functions and social behavior. CR has been observed to have negative effects on cognitive functions, probably caused by lower glucose levels (16), and on maternal care, inducing a decline in maternal behavior toward pups (17). However, CR also has positive effects, such as heightened social behavior between mice (18).

Typically, CR experiments require extended periods of time. Body mass control and avoiding potentially aggressive behavior (19–21) can force researchers to use single housing for animals. Especially if the aggressive mice behavior is considered (22). Single housing can cause many logistical issues (it requires more cages and racks, space and maintenance staff, etc.) as well as problems related to the well-being of animals (stress induction) (23). Regarding the logistical problems, current legislation [for a review see (24)] limits research installations and resources, discouraging individual maintenance of animals for extended periods of time. Single housing also impacts negatively on the animal's well-being [for a review see (25)], which could be a potential confounding variable in future protocols applied to animals in single housing. It is important to note that mice display complex social behavior such as empathy [for a review see (26, 27)], and the social deprivation associated with single housing in CR research may therefore have severe effects on the animal's behavior, by denying the animal access–for example–to the various benefits of social interaction. In this sense, social interaction has been shown to improve memory processes, reduce hippocampal damage in aged mice (28), induce brain-derived neurotrophic factor (29), reduce the impact of CR (30), induce higher food consumption (31), reduce anorexic behavior in adolescent mice (32) and facilitate cognitive recovery after a social defeat experience (33). All these aspects show the potential benefits of carrying out abundant research into CR with grouped animals. The benefits of grouping animals are not only logistical and economic, they are also very much related to their well-being, as described above.

Previous research in our laboratory showed the viability of group-housing while sustaining CR for long periods in male rats (34). Our results indicated the effectiveness of CR in different groups, regardless of the relationship between the rats. No extreme body mass changes were observed in CR rats, nor did they display aggressive behavior or show alterations in their corticosterone levels. To our knowledge, no similar data has been reported about the possibility of group-housing mice under CR. We decided to study CR in CB6F1/J mice for two reasons: it is a inbred strain often used in experimental research (35) and has a tendency to show aggressive behavior under grouped conditions (22). Our main objective was to determine how CR at 70% of *ad libitum* affects the body mass, relationships and behavior of littermates and unrelated (male or female) grouped mice, as well as to study as an indicator of stress, in relation to animal welfare (36, 37) by analyzing serum adrenocorticotropic hormone (ACTH) levels. It is expected that against aggressive behavior in C57BL/6 mice (22), a normal interaction with absence of significant aggressive behaviors will be observed in the CR at 70% mice. Likewise, we expected to find no significant differences in serum ACTH levels between the groups.

## Materials and Methods

### Ethical Approval and Other Ethical Considerations

Animals were kept in accordance with EU Directive 2010/63/EU and Spanish Royal Decree 53/2013. The University of Granada's Research Ethics Committee and the Junta de Andalucía, Consejería de Agricultura, Ganadería, Pesca y Desarrollo Sostenible approved the experimental protocol with reference number 09/08/2019/137. All animal procedures carried out for this study were subject to review by Animal-Welfare Officials and a designated veterinarian of the Animal Facility at CIBM/UGR.

### Animals

The experiment used fifty-four CB6F1/J (#100007; F1 generation hybrids from breeding of BALB/cJ females and C57BL/6J males from Jackson Laboratories) mice (27 female) from the Biomedical Investigation Center (CIBM) of Granada. Mice were divided into groups of three and kept in transparent methacrylate cages (215 × 465 × 145 mm) in rooms at 22 ± 1°C and with a 12-h light/darkness cycle (lights on at 7:30 AM). Standard Type-II Tecniplast LTD 370 cm^2^ cuvettes -allowing a maximum of 5 mice- were used for the maintenance of mice in the experimental phase, with pine wood shavings from Rettenmair Ltd, and enrichment elements (pieces of paper). This is the usual size used in the experimental maintenance of chronic and subchronic mice at the SPEA/IC/CI/CIBM facilities. The experimental subjects came from a 10 monogamous breeding pairs, in which the offspring were separated into groups of three after weaning in separate cages into males and females of the same litter, with *ad libitum* feeding for the littermate groups. For the unrelated groups, males and females were randomly selected from the cages of unrelated individuals. At the beginning of the experiment, the average body mass was 20.9 ± 1.13 g for females and 27.2 ± 1.53 g for males. Throughout the 23 days of the experiment, water was accessible *ad libitum* and a standard laboratory pellet diet (Harlan Teklad Research diet, Madison, WI, USA) was administered as described in the “Method” section below.

### Method

Cages with CR and *ad libitum* groups had the same body mass proportions and housekeeping conditions. To control the effectiveness of the restriction process, 18 unrelated mice (Group *ad libitum*; nine females and nine males housed in groups of three mice per cage) were designated as control groups. Since there was an absence of interaction during feeding in these unrelated controls groups, we consider not necessary to include another 18 littermate mice control group. Thirty-six mice (18 females and 18 males) were exposed to 70% food restriction. Each cage held a group of three mice, and they were distributed into four groups. In two groups, 18 unrelated mice (Group *Restricted unrelated; nine females and nine males*) were subjected to 70% food restriction, and 70% food restriction was also introduced in the other two groups of nine littermates mice (Group *Restricted-Related; nine females and nine males*). The tails of mice from each cage were marked in different colors (red, blue or no mark) to identify them.

### Recording Body Mass and Observing Behavior

Every day at 1:00 p.m., each mouse was weighed on a scale and food was administered. The *ad libitum* group was given 200 g of food, and groups on food restriction were given 70% of the food eaten each day by the *ad libitum* group of mice (The uneaten food from the *ad libitum* group was weighed). The remaining food (pellets) was removed before the CR groups had access to their food, to ensure there was a 70% food reduction. The order in which food was administered was rotated each day, thus producing parity between groups with regard to the time that mice had to wait for food (and the resulting added stress). After calculating the mean and standard deviation from the recorded body mass the body mass proportion between the three animals in each cage was calculated by considering the weight of the heaviest cage-mate mouse as 100% and applying the following equation:

$$\begin{array}{l} \%\;Body\;Mass=\frac{mouse\;weight\;\left(g\right)\;*\;100}{heaviest\;mouse\;weight\left(g\right)} \end{array}$$

The greater differences between mice weight, the lower the average % Body mass per cage.

Animals were recorded in their respective cages for 15 min every day, using a digital JVC camera model Everio HDD GZ-MG680BE, immediately after making the food available to both groups under CR. Of that time, the first 5 min were used to analyze behavior. At the end of the experimental procedure, a global analysis of social behavior was performed with the recorded material by using Behavioral Observation Research Interactive Software (BORIS) (38). Based on data obtained with rats in past research (34), the behavioral analysis focused on the number of times each mouse *pushed* its cage companions while eating. This *push* action can be compared to wrestling behavior observed in other studies (39, 40); a form of defense from other mice, using the front or back paws to indicate fighting behavior, or an attempt to force the mouse who is receiving the *push* to submit (submission response) (41). However, our previous results with rats (34) showed that *pushes*, under CR conditions, can be interpreted as social behavior. Also, potentially aggressive behavior (26) was recorded during the 15 min after food was made available to CR mice. Whenever such behavior was observed, the cage was eliminated from the experiment and CR was suppressed to prevent physical injuries.

In addition to the measurement of pushes, two additional behavioral from the total of 15 min recorded were analyzed. On the one hand, and within the aggressive behaviors, there was the recording of chasing behavior, understood as the time in which at least two mice chased each other to get a piece of food. On the other hand, and as part of the recording of more social behaviors, we measured the time in which the three animals were eating at the same time under the feeder without the presence of pushing and shoving. There were no other significant behavior to analyze.

### Hormonal Analysis

On the 23rd day of the experiment, after being deeply anesthetized with isoflurane, fifty microliters of blood were extracted intracardially from the 44 mice (10 samples were not collected due to the elimination of the Unrelated Male CR (9 samples) and the insufficient volume of blood for one female from the Unrelated CR group). To acclimatize mice to the procedure, they were previously exposed to the isoflurane box on two occasions. After the blood draw, animals were sacrificed with a cervical dislocation. Of the 44 mice, 18 were unrelated mice with food *ad libitum* (nine females and nine males), and another eight were unrelated, female mice on 70% food restriction. The remaining 18 were littermates on 70% food restriction (nine females and nine males). Serum was obtained from these blood samples for the hormonal analysis. Hormonal analyses were performed using the Milliplex map pituitary magnetic bead panel kit (MPTMAG-49K-01) for ACTH and the Luminex 200TM HTS, FLEXMAP 3D. Preparation of serum samples was performed as follows: blood was allowed to clot for at least 30 min before centrifugation, for 10 min at 1,000 x g. Serum was removed and assayed immediately. For each serum sample, 150 μl of the antibody-bead and assay buffer were added to the mixing bottle, resulting in a total volume of 2,850 μl. Next, samples were incubated overnight on a shaker at 4°C. Samples were then measured on the Lumina 200TM. Median Fluorescent Intensity (MFI) was recorded using a weighted 5-parametrer logistic or spline curve-fitting method to analyze concentrations in samples. The validation of the measurements made on the hematology counter was performed with a commercial artificial blood. Specifically, Myt-5D Hematology controls (normal control) from ORPHEE SA (CH-1228 Geneva/Pla-les-Quates SWITZERLAND) were used.

### Statistical Analysis

Statistical analyses were performed using JASP version 0.10.2. Behavioral data were analyzed considering the cage-mate mice as non-independents. Rest of the analyses were done considering each mouse as independent. Body mass and body mass proportion were compared using repeated measures (RM) Analyses of Variance (ANOVA) and Chasing behavior and Time under the feeder were analyzed by one-way ANOVA since the normality assumptions (sphericity and the equality of variances) were respected. Kruskal-Wallis non-parametric analysis was applied for ACTH levels and Total number of *Pushes* since the criteria of normality were not assumed in these cases. Whenever a significant difference between groups was found, the Bonferroni correction was applied in a *post-hoc* derived from the main analysis. The significance level in all cases was *p* < 0.05.

## Results

On the second day of CR, intensely aggressive behavior (26) was observed in two cages (six mice) of the group *Restricted unrelated* males. There was fierce fighting between animals, with blood and several skin injuries. This forced us to apply the ethical protocol and, as was mentioned above under *Method*, the nine animals of the group *Restricted unrelated* males were discarded from the experiment and CR was interrupted to avoid further fighting and possible injuries to mice.

### Body Mass and Body Mass Proportion

**Figure 1:**
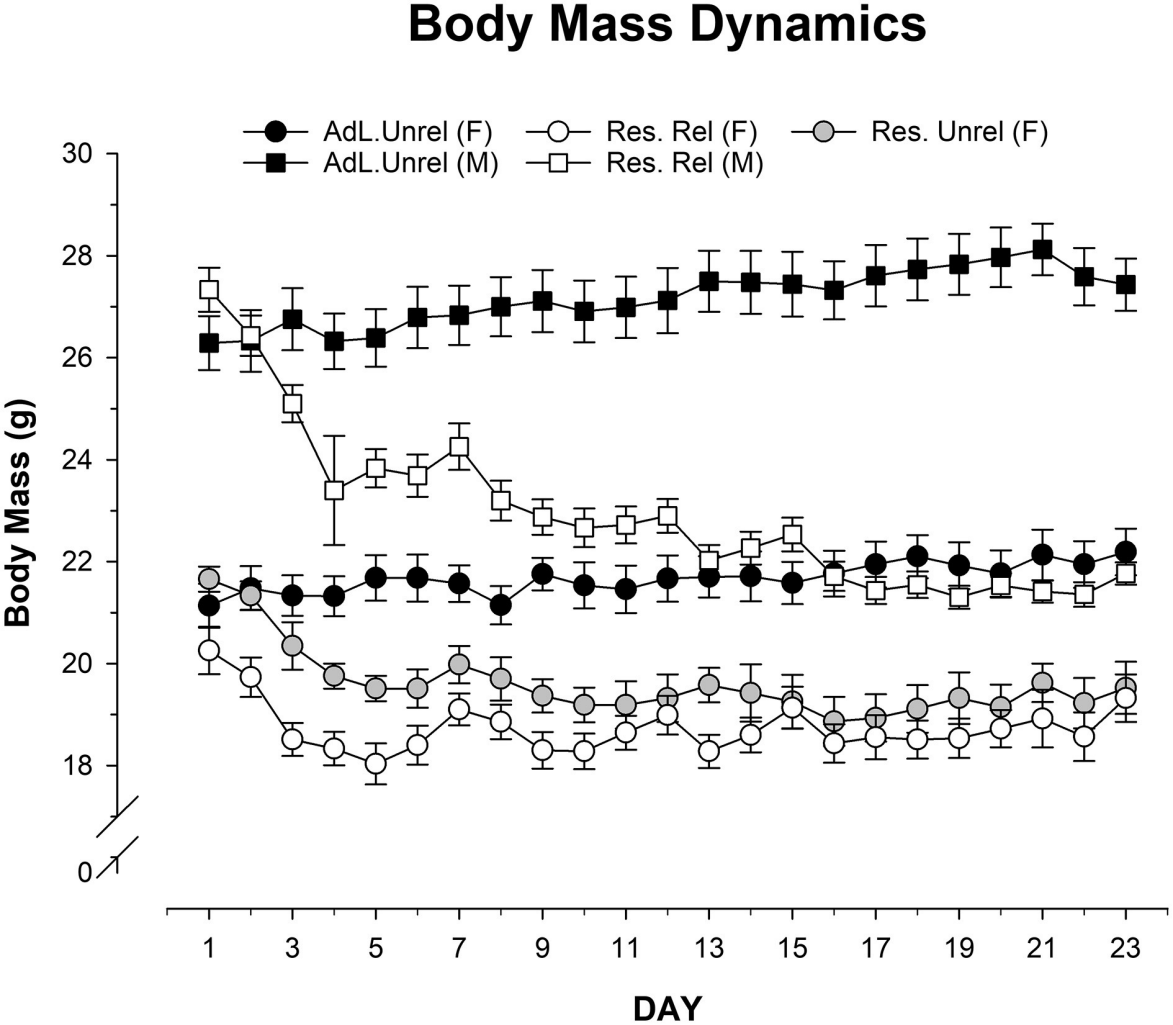
Mean (±SEM) body mass (g) throughout the 23 days (D) in *ad libitum* unrelated female and male (Adl.Unrel.), restricted unrelated female (Res. Unrel.) and restricted related female and male (Res.Rel) mice. Results showed the significant main effect of the variable group and the interaction between group and day. However, the variable day did not exert a significant main effect. Analysis of the interaction (keeping the first day as a covariate factor) shows that *ad libitum* Females have a significant higher body mass than restricted related female (days 3–23), restricted unrelated female and restricted related male (days 4–23). *Ad libitum* males have a significant higher body mass than restricted related female (days 3, 5, 6–14, 16–23). *Ad libitum* males have a significant higher body mass than restricted unrelated female and restricted related male (days 3, 5–23). Restricted related male have a significant less body mass than restricted related female (day 15, 17, 21). Restricted related male have a significant less body mass than restricted unrelated female (days 19, 21).

Figure 1 shows body masses for the five groups of mice (*ad libitum unrelated male and female, Restricted unrelated female, and Restricted related male and female*) throughout the 23 days of the experiment (for statistical analyses, first day was treated as a covariate factor). Application of RM-ANOVA here confirms the sphericity and equality of variances (Levene Fs < 2.03, df = 42; *p* > 0.09). Results showed a significant main effect of Group (ANOVA Between Subjects Effect: F = 131.517, df = 5; *p* = 0.001; η^2^ = 0.79) and the interaction between Group and Day (ANOVA Within Subjects Effect: F = 15.171, df = 105; *p* = 0.001; η^2^ = 0.078). However, the variable Day did not exert significant main effect (ANOVA Within Subjects Effect: F = 1.553, df = 21; *p* = 0.054; η^2^ Magnitude of the effect = 0.002). Analysis of the interaction (keeping the first day as a covariate factor) shows that *ad libitum* Females have a significant higher body mass than Restricted related Female since D3 to D23 (Bonferroni *p* = 0.001), Restricted unrelated Female since D4 to D23 (Bonferroni *p* = 0.001) and Restricted related Male D4 (Bonferroni *p* = 0.001), D5 (Bonferroni *p* = 0.002) and since D6 to D23 (Bonferroni *p* = 0.001). *Ad libitum* Males have a significant higher body mass than Restricted related Female D3 (Bonferroni *p* = 0.003), D5 (Bonferroni *p* = 0.002), and since D6 to D23 (Bonferroni *p* = 0.001) except D15 (Bonferroni *p* = 0.079). *Ad libitum* Males have a significant higher body mass than Restricted unrelated Female and Restricted related Male D3 (Bonferroni *p* = 0.011), and since D5 to D23 (Bonferroni *p* = 0.001). Restricted related Male have a significant less body mass than Restricted related Female Days D15 (Bonferroni *p* = 0.04), D17 (Bonferroni *p* = 0.02) and D21 (Bonferroni *p* = 0.006). The rest of the days no differences between these groups were significant. Restricted related Male have a significant less body mass than Restricted unrelated Female only days D19 (Bonferroni *p* = 0.048) and D21 (Bonferroni *p* = 0.005). Finally, Restricted related Female and Restricted unrelated Female did not have any significant difference in their body mass. This result shows the effectiveness of CR in male and female mice.

Regarding body mass proportions, after first checked the sphericity and equality of variances (Levene D1-D23 Fs < 2.178; *p* > 0.08), RM ANOVA showed that Group (ANOVA Between groups effect: F = 0.315, df = 5; *p* = 0.832; η^2^ = 0.02) and Day (ANOVA Within groups effect: F = 0.315; df = 22; *p* = 0.401; η^2^ = 0.004) variables had no effect, and nor did their interaction (F = 0.286; df = 110; *p* > 0.8; η^2^ = 0.019). This means that body mass proportions were similar throughout the days of the experiment.

### Pushes

**Figure 2:**
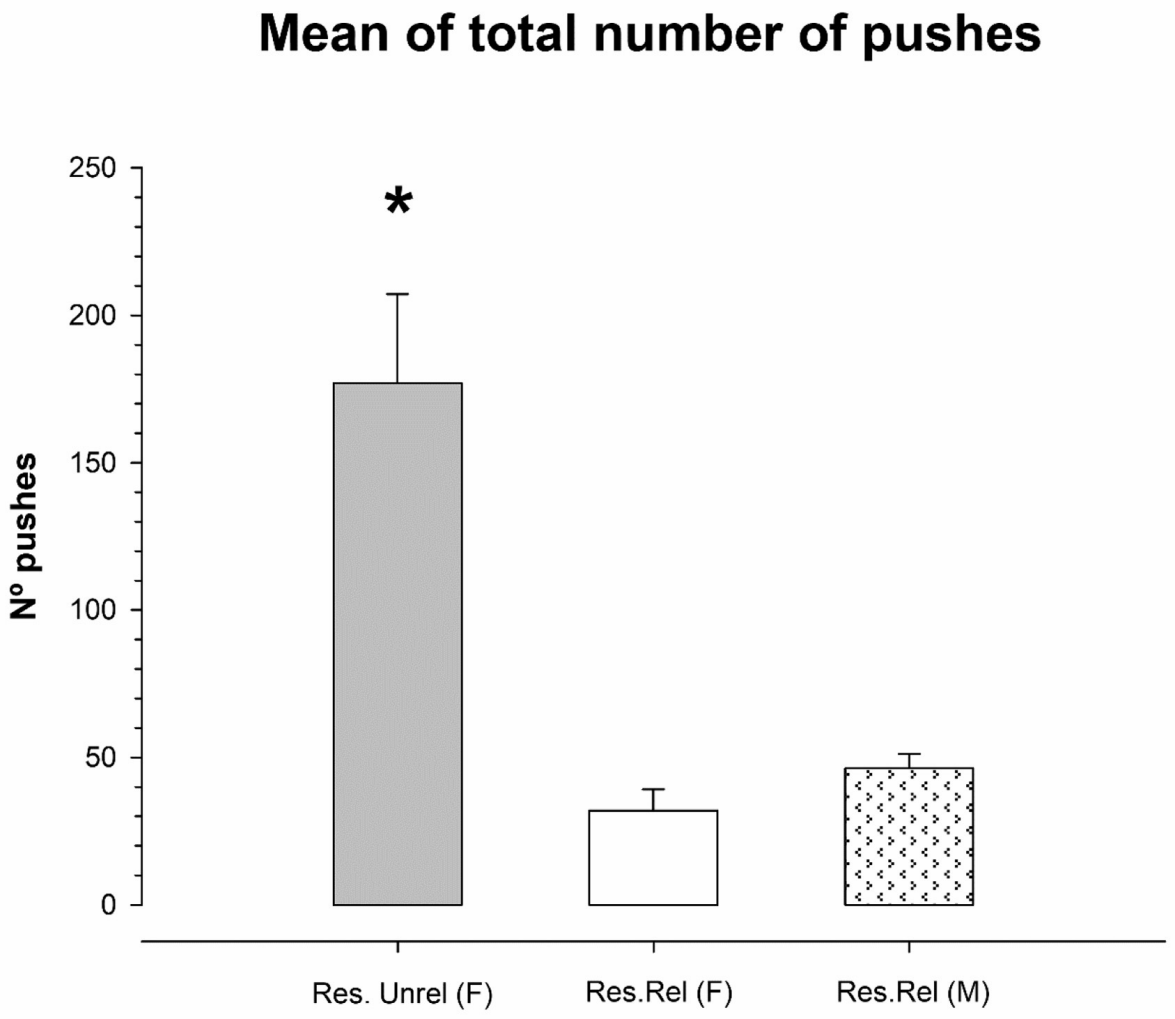
Mean (±SEM) of total number of *pushes* during the 23 days in restricted unrelated female (Res.Unrel) and restricted related female and male (Res Rel) mice. Restricted unrelated females showed a higher number of *pushes* than restricted related females and males as indicated by (*) sign.

Figure 2 shows the number of *pushes* under the feeder for *Restricted* unrelated and *Restricted related* mice. Due to the total absence of activity under the feeder when food was administered, the *ad libitum* groups were eliminated for analysis. A one-way ANOVA of the mean of the sum of pushes for each group (Unrelated female, Related female and Related males) throughout the days of the experiment showed a violation of the equality of variances (Levene F = 6.557; *p* < 0.04). The Kruskal-Wallis analysis showed significant differences between groups (K-W = 6.489, df = 5; *p* < 0.04). Comparing groups while applying the Kruskal-Wallis analysis shows a higher number of *pushes* in the group of unrelated females than in Related females and Related males (K-W = 3.857, df = 2; *p* = 0.05). No differences were found in the number of *pushes* between Related females and Related males (K-W = 2.33, df = 2; *p* = 0.12). Subsequent analysis of the cumulative frequencies of the pushes over the days showed for Restricted unrelated females (*p* = 0.029) and Restricted related males (*p* = 0.005) a significant linear component was observed (no stabilization of the number of pushes over the days). Quadratic or cubic component was not significant (*p* > 0.09). However, this linear, quadratic or cubic component was not significant for the group of sister females Restricted related females (*p* > 0.06).

### Chasing Behavior and Time Under the Feeder

Analysis of the total chasing behavior through the 23 days was done for Restricted unrelated and Restricted related mice after the absence of activity in the *ad libitum* groups. Application of ANOVA here confirms the sphericity and equality of variances (Levene F = 0.703, df = 2; *p* = 0.532). Analysis showed no significant effect of Group (One Way ANOVA: F = 0.290, df = 2; *p* = 0.748. η^2^ = 0.088). Similar, for the time under the feeder was done for Restricted unrelated and Restricted related mice. Due to the total absence of activity under the feeder when food was administered, the *ad libitum* groups were eliminated for analysis. Application of ANOVA here confirms the sphericity and equality of variances (Levene F = 1.697, df = 2; *p* = 0.261). Analysis showed no significant effect of group (One Way ANOVA: F = 1.395, df = 2; *p* = 0.318. η^2^ = 0.318).

### Adrenocorticotropic Hormone Levels

**Figure 3:**
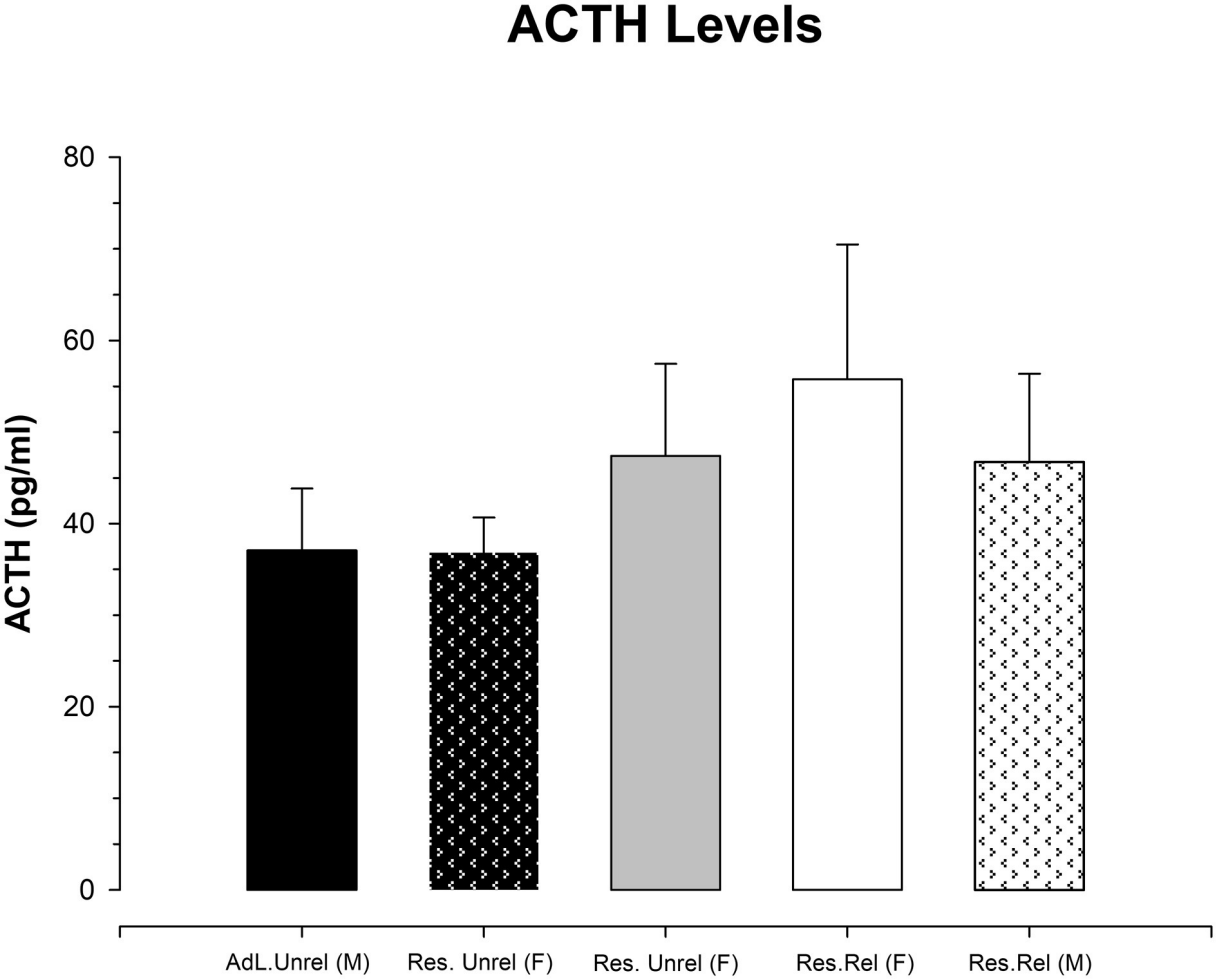
Mean (±SEM) of adrenocorticotropic hormone concentration (pg/mL) in *ad libitum* unrelated female and male (Adl.Unrel), restricted unrelated female (Res Unrel) and restricted related female and male (Res.Rel) mice collected on the 23rd day of the experiment. No significant differences between groups are observed.

Figure 3 shows the adrenocorticotropic hormone values obtained for the five groups. A one-way ANOVA revealed the violation of the equality of variances (Levene F = 3.329; *p* < 0.03). Kruskal-Wallis analysis shows the absence of significant differences between groups (K-W = 0.721, df = 5; *p* > 0.9). Additional analysis of the magnitude of the effect using the Cohen's *d* shows values between the groups negative or under 0.2 (lower effect). Only the differences between the restricted related female with the restricted related male and restricted no-related female had a Cohen's *d* of 0.242 and 0.221, respectively. This means that the CR procedure produced no significant increases in adrenocorticotropic hormone levels in any group.

## Discussion

The first result that requires comment is the impossibility of applying CR in unrelated male mice, contrary to the lack of aggressive behavior observed in unrelated rats under CR conditions (34). This data supports the greater aggressiveness previously observed in mice (42), and particularly in BALB/C mice (22, 43, 44). This greater aggressiveness in males toward other males (45) made CR impossible among unrelated males. However, in littermates, and in unrelated female mice the aggressiveness does not appear and neither does clear a type of considered cooperative social behavior such as the time under feeder of all the animals at the same time. This possible absence of social behavior (measured in our experimental conditions) can also be interpreted as a sign of less complex empathy in mice. Although mice display empathy (27) and social behavior (46), it seems that under CR conditions this social and cooperative behavior does not appear even though the mice lived together after weaning for 7–8 weeks. Groups of littermates showed lower levels of aggressiveness, confirming previous observations in mice (47) and this was demonstrated in the non-aggressive behavior observed under CR conditions in male and female mice. In the case of unrelated females, the correct development of CR conditions was facilitated by levels of aggressiveness that were lower than usual among non-pregnant females (45). However, an analysis of the *pushes*, shows a higher total number of pushes in unrelated female mice under CR conditions than in related female and male mice. This was an unexpected result. In previous research, aggressiveness implies another kind of behaviors more violent (attacks, bites or squeaks). In our experiment, this kind of behavior was only showed in the restricted unrelated mice, but was not observed in the rest of the restricted groups. Only chasing behavior was observed and the differences between groups were no significant between restricted unrelated female and the restricted female and male littermates. Differences were observed only in pushing. The *pushes* observed in unrelated female mice did not imply aggressive behavior, causing (sometimes) other mice to shuffle or fall. Perhaps this low aggressive behavior is explained considering that it was observed in females. Another possible explanation for this behavior could be the 7–8 weeks that unrelated female mice were housing together. This previous cohabitation and the grouping right after weaning might perhaps mitigate this aggressive response that have been observed in restricted unrelated males (47). These data open the possibility to considerer other variables such as time of cohabitation apart and not only the strain or the characteristics of grouping (22, 35). Likewise, consideration of the possible influence of environmental changes on the induction of aggressive behaviors (e.g., as the observed in transportation to research facilities) or housing conditions (42) should be noted.

These positive effects have been observed in the adrenocorticotropic hormone analysis. An absence of significant differences between groups, which could be interpreted as an indirect measure of the absence of alterations in stress levels (36, 37, 48). It is also true that samples were taken at the end of 23 days under CR conditions, and these levels could therefore actually be associated with other biomarkers. In this respect, the possible role played by orexin has been studied as a neuropeptide that might connect prolonged food restriction periods, aggressiveness and social behavior (46, 49–51). The long time period between the CR and the adrenocorticotropic measure has perhaps have produced an adaptation as probably other biomarkers such as feeding times, usually done in the dark period [for a review see (52)]. It might be interesting to further investigate in this area, to clarify not only the aggressive response associated to CR in unrelated mice, but also changes in biomarkers. However, in our study the objective was just to evaluate the viability of applying long CR in mice despite its inherent aggressiveness (22).

Lastly, and regarding the effectiveness of CR, while there was a 21% reduction in body mass in related males under CR relative to *ad libitum* males, which is consistent with previous results in rats (34, 53, 54), body mass reduction associated with CR in females was lower (13%). This result can be attributed to differences observed between female and male metabolism (55, 56).

## Conclusions

Our results have important implications, particularly in relation to the difficulties attached to long-term CR in unrelated male mice. In that context, and based on the results observed in terms of aggressiveness, the best option when implementing CR in mice would be to group-house littermates.

## Data Availability Statement

The raw data supporting the conclusions of this article will be made available by the authors, without undue reservation.

## Ethics Statement

The animal study was reviewed and approved by The University of Granada's Research Ethics Committee and the Junta de Andalucía, Consejería de Agricultura, Ganadería, Pesca y Desarrollo Sostenible approved the experimental protocol with reference number 09/08/2019/137.

## Author Contributions

CP, IM, JZ, and CC: study concept and design. CP and IM: acquisition of data. CP, LR-L, AV-Á, and IM: analysis and interpretation of data and statistical analysis. IM: drafting of the manuscript. CP, LR-L, AV-Á, IM, JZ, and CC: critical revision of the article for important intellectual content. JZ and CC: obtained funding. All authors had full access to all the data in the study and take responsibility for the integrity of the data and the accuracy of the data analysis.

## Conflict of Interest

The authors declare that the research was conducted in the absence of any commercial or financial relationships that could be construed as a potential conflict of interest.
